# Healthcare Professionals’ Perceptions of Medicine Shortages in Public Health Facilities of the Eastern Cape, South Africa

**DOI:** 10.3390/healthcare14050683

**Published:** 2026-03-08

**Authors:** Mmabatho Miriam Ndwandwe, Mygirl Pearl Lowane, Thembi Simbeni, Mathildah Mpata Mokgatle

**Affiliations:** Department of Public Health, School of Healthcare Science, Sefako Makgatho Health Sciences University, Pretoria 0204, South Africa

**Keywords:** medicine shortage, healthcare professionals, perceptions, public health facilities, supply chain management, South Africa, pharmaceutical governance

## Abstract

Background: Medicine shortages present a critical challenge for health systems worldwide, impacting patient care and health outcomes. This study investigated healthcare professionals’ perceptions of the impact of medicine shortages in public health facilities of the Eastern Cape, South Africa. Methods: A quantitative, cross-sectional design was employed, using a self-administered questionnaire distributed to 394 healthcare professionals (professional nurses, pharmacists, and medical officers). Results: The findings revealed a strong consensus that shortages negatively affect all stakeholders, with 96.7% of respondents agreeing that they increase provider stress and reduce patient trust. A significant majority also reported that shortages lead to delayed treatment (70.6%) and compromised health outcomes (67%). However, perceptions varied significantly by profession. Pharmacists were significantly more likely than nurses and medical officers to perceive that shortages result in increased out-of-pocket costs for patients, treatment errors, and compromised health outcomes. Conclusions: The study concludes that medicine shortages severely impact patient safety, clinical outcomes, and healthcare providers’ well-being in this setting. The pronounced concerns among pharmacists highlight their strained role in managing the crisis and underscore the urgent need for strengthened pharmaceutical supply chain governance, interprofessional collaboration, and targeted policies to mitigate the effects of shortages and protect patients from financial hardship.

## 1. Introduction and Background

In recent years, all countries have experienced an increase in shortages of medical supplies. The World Health Organization (WHO) reports that low-, middle-, and high-income countries worldwide are experiencing shortages of basic medicines [1]. The most affected countries are high-income countries [1]. Access to affordable, essential medicines is one of the objectives of Sustainable Development Goal (SDG) 3.8 [2].

According to the WHO, medicine shortages are situations in which the supply of medicines, vaccines or other health products needed to meet public or patient needs is insufficient [1]. Many definitions vary depending on the aspects of the supply chain to be addressed. For example, the definitions for manufacturing-related problems differ from those for patient distribution. The National Medicine Regulatory Authority (NMRA) defines the requirement for reports on shortages as a situation in which manufacturers or importers of medicines are unable to meet current or planned demand, including temporary disruptions or permanent interruptions in the production and supply of medicines [3].

Patients are dependent on medicines to improve clinical outcomes. When medicines are not available when needed, they are defined as shortages of medicines [4]. This definition uses a time factor or inability to supply patients within a certain period [5,6]. The shortage of medicines is reported as soon as the market become aware that the continuous supply of medicines, as required, will not meet the expected patient demand for more than 14 days [7,8]. The lack of appropriate medicines for patient treatment has led to many other challenges, such as the need to substitute medicines, patients not receiving treatment, and the development of complications [9], which may require re-admission to hospitals or long-distance referrals to hospitals that can treat them.

Previous research has consistently shown that healthcare professionals experience medicine shortages as an important public health issue impacting patients, clinical practice, and the efficiency of health systems [10]. For example, an enormous amount of labour and resources is required to address shortages [11], where nurses, pharmacists, and medical officers work longer hours and shifts and lack support. These factors create unsafe working conditions when these workers are physically, mentally, and emotionally exhausted and can unintentionally affect the treatment of patients [12]. International studies have shown that a shortage of medicines leads to deviations from standard treatment guidelines, delays the introduction of treatment, increases the risk of medication errors, and increases the cognitive and administrative burden of front-line staff [4,13,14]. These effects are particularly pronounced in hospitals where shortages of essential and life-saving medicines are common [15]. Research highlights that pharmacists and physicians are sometimes compelled to use less familiar or less effective alternative medicines due to shortages [6,16]. This situation generates frustration and moral distress, as healthcare professionals face ethical dilemmas arising from conflicts between personal, professional, institutional, and societal values, and they are caught between maintaining standards of care and managing limited resources [16].

There are many contributing factors to medicine shortages across different countries and regions. South African public hospitals are also facing this ongoing medicine shortage, which poses a threat to government plans to ensure universal health coverage for all [17,18]. The shortage of medicines in South Africa’s public health sector affects various levels of care, including clinics, community health centres, district hospitals, regional hospitals, tertiary hospitals, and central hospitals [19,20,21]. Due to the complexity of this problem, government agencies have been working with associations and institutions to develop policies, guidelines, and programmes to address the unavailability of medicine. Each country’s economic stability and strong administration play an important role in developing strategies to address and eliminate medicine shortages. Countries with a solid research capability and the financial capacity to support health system initiatives can address the shortage of medicines [22,23] and accelerate the approval of medicines manufactured by the relevant regulatory authorities [24,25].

Since the causes of the lack of medication vary from country to country, there are currently no standard interventions. The management of the shortage of medicines is heavily dependent on the health system, which invests significant resources to establish a model for managing, monitoring, and frequently reviewing the pharmaceutical supply chain [21,26,27].

The shortage of medicines has been widely documented worldwide, including in South Africa at the national level. There is limited quantitative evidence in the province of Eastern Cape that captures how different categories of healthcare professionals in the public sector perceive and experience these shortages. Most of the existing literature is based on national analyses, qualitative accounting, or facility-specific reports, leaving a gap in empirically based, profession-disaggregated data in one of South Africa’s most resource-limited provinces. Medicine stock-outs are a national crisis, and literature suggests that the Eastern Cape has felt the effects even more in the last few years. Data collected by the Stop Stock-Outs Project (SSP) in the province indicates that the total number of stock-outs recorded between April and October 2020 was approximately double the figure reported for the corresponding period in 2019 [28]. Poor management has been identified as a significant contributor to medicine stock-outs in health facilities within the province of Eastern Cape. The availability of adequate pharmaceutical supplies is essential for ensuring that healthcare professionals, including doctors and nurses, can deliver treatment efficiently and effectively, while minimising the risk of treatment interruption or default.

The study addressed this gap by providing specific quantitative evidence on the impact of medicine shortages in the Eastern Cape public health system. By comparing perceptions across professional categories, we go beyond descriptive reporting of stock-outs and provide an overview of how roles, responsibilities, and the proximity to medicine management shape the experience of the healthcare professionals responsible for medicine governance. In this regard, the study generates evidence-based information to inform policy formulation by identifying systemic shortcomings in stock-out control, communication, and accountability structures. The findings provide a basis for strengthening medicine supply chain policy, refining the stock monitoring framework and directing targeted interventions to enhance the supply of medicine in public sector facilities. Ultimately, the study provides data-driven insight to support more responsive and sustainable pharmaceutical governance in the public health sector. The study thus investigated the opinion of healthcare professionals on medicine shortage in public health sector facilities, with the aim of mitigating the challenge.

## 2. Methods

### 2.1. Research Design

A quantitative cross-sectional descriptive design was used. This approach was adopted because it provides a snapshot of healthcare professionals’ perceptions and the impact of poor medicine governance in public sector health facilities in the province of Eastern Cape. The approach was structured in line with the WHO Good Governance for Medicines (GGM): Model Framework.

### 2.2. Study Setting

The study took place at public sector health facilities of the Eastern Cape Department of Health in the province of Eastern Cape of South Africa. The Eastern Cape health system operates mainly in a rural environment characterised by dispersed settlements, inadequate transport infrastructure, and facility constraints that directly affect the distribution, storage, and delivery of medicines [29,30]. Despite the operation of two pharmaceutical depots, the province is still experiencing persistent and recurrent shortages of medicines, making it a critical point for examining systemic weaknesses in procurement, governance and accountability [31].

The province is divided into three regions: Eastern (Mthatha), Central (East London) and Western (Port Elizabeth) regions. Each region has clinics, community health centres, two regional hospitals, and a tertiary hospital. The study setting covers all levels of care. Moreover, the selection of the province of Eastern Cape was based on its distinctive structural, geographical, and health system characteristics that render it a critical and information-rich context for examining medicines governance and shortages within the public sector.

### 2.3. Target Population and Sampling

The target population involved public healthcare employees, i.e., professional nurses, pharmacists, and medical officers working at different levels of care, i.e., primary (PHC), secondary (regional hospitals), tertiary (tertiary hospitals), and quaternary (central/academic hospitals) healthcare services. Respondents were at least 18 years old and had been practising in the designated profession for more than a year. They must be registered with their respective statutory bodies.

There were about 5300 public healthcare employees working at different levels of healthcare service delivery, as found in the payroll of 2024 of the province of Eastern Cape. These employees were spread to over 711 clinics, 28 community health centres, and 92 hospitals. A one-stage probability cluster sampling design was used. Cluster sampling is a probability sampling method that divides a population into mutually exclusive natural groups and then selects an entire cluster for incorporation, not the entire population [32]. The selection strategy took into account the wide geographical distribution of the province and ensured a wide scope of coverage that was comprehensive to all levels. In this study, public health facilities were the main sampling units (clusters). The facilities chosen to represent different levels of care in the Eastern Cape public health system include primary health clinics, community health centres, and hospitals. The clusters were as follows: The Eastern Cape Department of Health consists of three administrative regions. Each region is organised to fully support the entire process of referral, from primary health facilities, regional hospitals and tertiary hospitals, to provide specialised services. This approach represents a one-step probability cluster sampling design in which facilities form the main sampling units, not individual health workers. The first area was a tertiary hospital, the second area was a regional hospital, and the third area was a primary healthcare platform (clinics, community health centres and district hospitals).

The selection of facilities as clusters reflects the functional organisation of health professionals and enables efficient data collection across a large, mainly rural province. All qualified health professionals, medical officers, professional nurses, and pharmacists, from the selected facilities were invited to participate, forming a one-step cluster design. This approach reduces logistical barriers, increases participation rates, and ensures that professionals are centrally represented in the management of medicines while maintaining the probability sampling principle.

The sample size was calculated using the Raosoft calculator from the population above, and the final sample size was estimated at 396, with a 95% confidence level, 5% margin of error, 50% population size, and a 10% buffer.

### 2.4. Recruitment of the Respondents

After receiving ethical approval from the university research committee, the researchers began recruiting the respondents for the study. The primary investigator wrote letters to the provincial Department of Health and the facilities for permission. The primary investigator also consulted with the facility manager to introduce herself to the staff and then introduce the study to interested individuals. An appointment to obtain informed consent and administer the questionnaire was arranged on that day.

### 2.5. Data Collection Instrument and Procedure

A self-administered questionnaire was used. This instrument was formulated based on the WHO GGM: Model Framework. It was divided into sub-sections, where Section A captured the socio-demographic characteristics of the respondents and other sections captured the perceptions and impact of poor medicines governance to achieve the study goal.

The questionnaires were given to respondents at the identified health facilities throughout the distinct levels of care to complete in the morning before the routine, during lunchtime, or after work. When distributing the questionnaires, informed consent was obtained, and respondents were asked to sign the consent forms, which were kept separate from the questionnaires. The respondents were requested to submit the completed questionnaires to the primary investigator in a sealed envelope provided by the primary investigator on the same day immediately after completion. If the principal investigator was unable to collect the completed questionnaires, the facility manager was asked to collect the questionnaires on behalf of the principal investigator. Data was collected between July and November 2024.

### 2.6. Validity, Piloting and Reliability of the Instrument

To ensure the instrument’s validity, the researchers assessed the data collection tool’s reliability prior to the data collection. A comprehensive literature search was done to ensure that the results of this study can be comparable with other studies with similar research objectives. Furthermore, to ensure content validity, pre-testing of the instrument was conducted before the main study commenced, allowing inappropriate or unclear questions to be corrected, rephrased, or discarded.

Piloting of the instrument was conducted among 20 respondents who were not part of the main study but were from the same population and met similar criteria. Thirty to fifty minutes were allocated to respondents to complete the questionnaire, and they were allowed to contribute where necessary. The principal investigator analysed the questionnaires to assess whether respondents had followed the instructions, checked completeness, assessed questions that respondents mostly skipped, and checked whether the time allocated to complete the questionnaire was realistic. The principal investigator then engaged the respondents after the exercise, and their comments were used to revise the questionnaire.

Reliability was observed by assessing the extent to which the findings captured were similar and valid to the extent that they addressed the same research problem to achieve the common goal among respondents. Reliability is referred to as the consistency and stability of the research instrument. After the tool’s modification was done to ensure reliability, the instrument’s test–retest was conducted among the same respondents. They were asked to complete the same amended questionnaire on two separate days. The results were analysed and compared using Cronbach’s alpha correlation technique to determine internal consistency and coherence of a set of items in an instrument. Values ranging between 0.6 and 0.7 are regarded as representing acceptable reliability, whereas values of 0.8 and above are regarded as good reliability indicators [33]. Cronbach’s alpha of 0.8 was obtained, and the results indicated that the items reliably measured the same basic concepts; the instrument was considered reliable, so the primary investigator concluded that the items could be used in the questionnaire.

### 2.7. Data Processing and Analysis

The completed questionnaires were scrutinised, and those that were found to lack more than 10% of the information were excluded. Missing data occurs when an observation has no recorded value. Within a dataset, no missing data is present when a value is entered for every input and output variable for each element [34]. Methodologically, the extent of missing data is commonly categorised as small, moderate, or significant. A missing data rate below 5% is often considered small and negligible, while a rate between 5 and 10% is generally acceptable, with minimal impact on results. In contrast, missing data exceeding 10% poses a significant threat to a study’s validity, reliability, and statistical power, increasing the risk of biased outcomes [34,35].

Data was captured in Microsoft Excel. Data was analysed using SPSS version 29 (2022). The analysis was done following descriptive and inferential statistics. The mean and standard deviation (SD) for each statement were calculated by weighting the response category on its scale. Frequencies and percentages were calculated to summarise healthcare professionals’ perceptions of the impact of medicine shortages. Because the perception items (agree, neutral, disagree) were measured on an ordinal scale, the Kruskal–Wallis (KW) test was used to assess differences in median perception scores across multicategory biographical variables such as age groups, years of service, profession, and facility level. The Kruskal–Wallis test is a non-parametric alternative to one-way ANOVA, suitable when normality assumptions are not met. In the questionnaire responses, neutral was considered a “silent” disagree, as it was interpreted that the respondents were unsure or did not understand the point under discussion.

The assumption that neutral is silent disagreement came from the psychometric and behavioural decision theory as well as the Likert measurement theory [36,37]. In perception research, neutral does not always represent a midpoint belief. The study was performed within the health system where the respondents were expected to state their perceptions in relation to the functionality of medicines governance. Assuming that neutral is a silent disagreement is not necessarily stating that neutral equals disagreement, but rather non-endorsement of the statement.

For instance: *“Medicine availability is included in the executive management key performance indicators”*

Agree—accepting that the statement is correct,

Neutral—lacking confidence about the statement,

Disagree—explicit rejection of the statement.

The assumption was leaning more on the asymmetric nature of the responses that a neutral response is not endorsing the perception as it is represented by the statement. Agree represents endorsement whereas neutral and disagreement represent lack of endorsement of the statement. Survey methodology literature further shows that midpoint selections frequently function as uncertainty, “don’t know,” or socially safe responses rather than true attitudinal neutrality, particularly in organisational environments [38,39,40]. Collapsing responses into agreement vs. non-agreement (neutral + disagree) is a standardised procedure when the research question concerns confidence in system functioning.

For variables with only two categories, such as gender, the chi-square test was used to examine associations with perception items. A *p*-value of <0.05 was considered statistically significant. Multinomial logistic regression analysis was used to examine associations between respondent characteristics and perceptions of the consequences of medicine shortages. Both crude and adjusted models were fitted. The adjusted models included gender, professional category, and facility level as covariates, selected a priori based on their established relevance in influencing healthcare practice and perceptions. Adjusted odds ratios with 95% confidence intervals are reported. The results of the study are shown in tables.

The selection of these statistical models was informed by the nature of the data collected. The perceptual results were measured on an ordinal scale of three points (disagree, neutral, and agree), and preliminary findings indicated that normal distribution assumptions were not met, so the non-parametric method was considered appropriate. The Kruskal–Wallis test compares the median perception scores of two independent groups (e.g., occupation, age group, facility level), while the chi-square test compares the association between two categories of variables. Multinomial logistic regression was used to study the relationship between the characteristics of respondents and the perception results of more than two independent categories, allowing the simultaneous comparison of neutral and agreement responses. The covariates included in the adjusted model (gender, professional category and facility level) were selected based on data collected and existing literature and their established relevance to the roles of medical practice, exposure to the management process of medicine, and differing experiences of a lack of medicine.

### 2.8. Ethical Considerations

Ethical clearance was obtained from the Sefako Makgatho University Research Ethical Committee (SMUREC/H/28/2024:PG). Permission to conduct the study was sought from the provincial and district Departments of Health in Eastern Cape, including the hospitals where the study was conducted. To obtain informed consent, the purpose of the study was explained to respondents in detail, and a consent form was given to each respondent to review and ask questions for clarity concerning the study. Upon agreement to participate, they were asked to sign the consent form. Confidentiality and autonomy were maintained through the codes used in the questionnaire. The respondents were informed of the research’s scope, including its benefits and potential risks. The main investigator allowed the respondents to complete the questionnaire in their own safe place, to protect them from psychological or physical harm, and ensured that the questionnaire addressed only the research objectives. To ensure the principles of justice, the same questions were asked regardless of their work positions. Respondents’ right to privacy and personal data were protected in accordance with the Protection of Personal Information Act no. 4 of 2013 (POPIA) (Staunton & De Stadler, 2019).

## 3. Results

### 3.1. Socio-Demographic Characteristics

Of 396 respondents, 394 were healthcare professionals from three healthcare professional categories, namely, professional nurses (217: 55.1%), pharmacists (100: 25.4%), and medical officers (77: 19.5%). Two candidates withdrew, resulting in a very high response rate of 99.5%. Many were female (281: 71.3%), there were 21 nurses (55.1%), and 199 were based at a hospital (50.5%).

The age and years of service were summarised using descriptive statistics, namely, minimum, maximum, lower (LQ) and upper quartile (UQ), mean, mode, and median. The youngest respondent was 28 and the oldest was 65. The lower and upper quartiles show that half of the respondents were between 41 and 53 years old, which indicates a quarter of them were younger than 41 and a quarter were older than 53. The average age was 46.9 years, and the median age was 47 years. With respect to years of service, the minimum was 2 years and the maximum was 40 years; half of them had been in the service for between 13 years and 23 years. The complete distribution is shown in the frequency table (Table 1).

### 3.2. The Perceptions of Healthcare Professionals on the Impact of Medicine Shortages

Table 2 presents the distribution of healthcare professionals’ perceptions regarding the impact of medicine shortages. Overall, most respondents strongly perceived medicine shortages as having significant negative consequences for patients and the health system. Nearly all respondents (93.4%) agreed that shortages affect all stakeholders, and 80.2% acknowledged that shortages place additional costs on patients across economic levels. A sizable proportion (70.6%) indicated that shortages result in delayed treatment, hospitalisation, or the use of substitution medicines.

Perceptions were more divided regarding financial implications and clinical errors. Less than half (48.7%) agreed that out-of-pocket costs increased due to shortages, while over one-third (36.3%) remained neutral. Similarly, only 38.6% agreed that shortages contribute to prescription or dispensing errors, with substantial neutrality (34.5%) and 26.9% disagreement, indicating mixed views.

In contrast, there was overwhelming agreement on the effects on healthcare providers’ well-being: 96.7% believed that shortages increase stress and frustration and reduce patient trust. Strong agreement was also observed regarding ethical dilemmas, with 74.6% noting that clinicians are often forced to ration medicines or switch to alternative therapies. Lastly, 67% agreed that shortages compromise health outcomes, including increased risk of re-admission, morbidity, and mortality.

### 3.3. Associations Between Perceptions and Categorical Biographical Characteristics

Table 3 shows the bivariate associations between healthcare professionals’ perceptions of medicine shortages and key demographic characteristics (age, years of service, gender, profession, and facility level). There were no significant associations between most perceptions and gender, age, or years of service. Professional and facility levels showed the strongest associations with multiple perception items. Profession was significantly associated with views on stakeholder impact (*p* = 0.015), extra costs for patients (*p* = 0.001), out-of-pocket costs (*p* = 0.001), treatment/dispensing errors (*p* = 0.001), denied or delayed treatment (*p* = 0.001), and compromised health outcomes (*p* = 0.001). This suggests differences in how medical officers, nurses and pharmacists observe shortages within their roles. Similarly, facility level (primary, district, tertiary) was significantly associated with perceptions of extra patient costs (*p* = 0.001), out-of-pocket costs (*p* = 0.001), treatment/dispensing errors (*p* = 0.037), and compromised health outcomes (*p* = 0.090, marginal).

### 3.4. The Out-of-Pocket Cost to Patients

Table 4 presents the adjusted associations between respondent characteristics and perceptions that medicine shortages increase patients’ out-of-pocket costs. Gender was not significantly associated with either neutrality or agreement regarding increased costs. Profession, however, showed a statistically significant relationship. Nurses were significantly less likely than pharmacists to agree that medicine shortages result in increased out-of-pocket costs (adjusted OR = 0.187; 95% CI: 0.061–0.578). Facility level also demonstrated a significant association, with staff working in clinics showing significantly lower odds of agreeing that shortages increase out-of-pocket costs compared with those working in hospitals (adjusted OR = 0.349; 95% CI: 0.141–0.867). No other adjusted associations reached statistical significance.

### 3.5. Medicines-Related Errors in Treatment, Prescribing, Dispensing, and Administration

As shown in Table 5, gender was not significantly associated with perceptions of treatment, prescription, dispensing, or medicine administration errors arising from medicine shortages. In contrast, profession was strongly and consistently associated with perceived errors. Both medical officers and nurses were significantly less likely than pharmacists to agree that medicine shortages lead to such errors, with nurses demonstrating particularly low odds of agreement (adjusted OR = 0.122; 95% CI: 0.052–0.286). Nurses were also significantly less likely to report neutrality regarding errors (adjusted OR = 0.165; 95% CI: 0.067–0.404). At the facility level, clinic-based staff had significantly higher odds of expressing neutrality than hospital-based staff (adjusted OR = 2.442; 95% CI: 1.157–5.155), suggesting greater uncertainty or variability in experiences of errors during shortages. No significant associations were observed for community health centres or for agreement at the clinic level after adjustment.

### 3.6. Deferred or Withheld Treatment, Prolonged Hospitalisation, and Adverse Medicine Reactions

Table 6 shows the adjusted associations between respondent characteristics and perceptions of delayed or denied treatment due to medicine shortages. Across gender, profession, and facility level, no statistically significant associations were observed. Although some odds ratios suggested increased or decreased likelihoods of neutrality or agreement, all corresponding confidence intervals were wide and crossed unity, indicating no reliable evidence of association. These findings suggest that perceptions of delayed or denied treatment were relatively consistent across demographic and professional groups.

### 3.7. Medicine Shortages Contribute to Adverse Health Outcomes

Table 7 summarises perceptions that medicine shortages compromise health outcomes, including morbidity, mortality, inappropriate substitutions, and hospital re-admissions. Gender was not significantly associated with either neutrality or agreement. Profession, however, showed statistically significant associations. Medical officers were significantly less likely than pharmacists to both express neutrality (adjusted OR = 0.109; 95% CI: 0.019–0.622) and agree (adjusted OR = 0.177; 95% CI: 0.036–0.861) that shortages compromise health outcomes. Similarly, nurses were significantly less likely than pharmacists to agree with this perception (adjusted OR = 0.128; 95% CI: 0.025–0.665). Although community health centre staff demonstrated elevated adjusted odds compared with hospital staff, the wide confidence intervals crossing unity indicate imprecision and lack of statistical significance. No clear or consistent facility-level pattern was observed after adjustment.

## 4. Discussion

The study provides critical insight into the perception of health professionals in the Eastern Cape, South Africa, on the impact of medicine shortages. The findings reveal a strong consensus that shortages have a serious negative impact, consistent with global research. The impact of medicine stock-outs on healthcare professionals cannot be underestimated [41]. The medicine shortage situation leaves healthcare providers dissatisfied, stressed, exasperated, and losing patients’ trust, which was rated highly by all healthcare professionals in the study. Increasing frustration between pharmacists and medical officers has been strongly documented in a United Kingdom (UK) policy report [42]. Pharmacists bear personal blame when the health facility experiences medicine shortages, as other healthcare professionals (medical officers and nurses) struggle to accept that the facility has run out of essential medicines [43]. These different feelings among healthcare professionals create tension that impairs interprofessional relationships. The high levels of stress and the possibility of deterioration of the interprofessional relationships highlighted in our study are crucial. This is consistent with a UK policy report documenting increasing frustration among pharmacists and medical officers and highlighting the need to improve cooperation and communication for joint planning [6,42]. Healthcare professionals often must ration medicines or use available substitutes, and this is an ethical dilemma shared worldwide, as seen in Mauritania and Pakistan [6,44].

One of the most important findings of this study is the pronounced differences in perceptions across professional roles. Pharmacists have always expressed a more pronounced understanding of adverse effects than nurses and medical officers. They were much more likely to agree that shortages result in increased patient expenditures, medication errors and deteriorating health outcomes. These findings indicate that pharmacists report medication-related errors more frequently than nurses and medical officers, possibly reflecting their direct involvement in the processes of medicine supply, verification, and dispensing. This is consistent with the literature; pharmacists are directly responsible for the supply chain and are often “personally responsible” if medicines are unavailable [43]. Our findings indicate that pharmacists report more errors, which aligns with a European study demonstrating that pharmacists’ direct involvement in distribution makes them more likely to detect and report prescription and administration errors resulting from substitutions [5]. In contrast, nurses and medical officers may perceive errors differently along the patient care pathway, and the direct link between stock-outs and the subsequent administrative error may not be apparent.

Pharmacists work at the interface of supply chain management and regulatory responsibility. They manage stock controls, comply with standard treatment guidelines and the Essential Medicine List, and report availability to management and monitor inventory levels and supplier performance in real time. As a result, they often first recognise shortages and tend to see them as preventable governance or procurement failures, rather than clinical disruptions. Meanwhile, medical officers face shortages at the clinical decision-making stage. They are aware of stock shortages when prescribed drugs cannot be dispensed, creating deviations from treatment guidelines. For them, a shortage of medicines poses a threat to clinical management and raises concerns about patient standards and outcomes. Lastly, professional nurses suffer a shortage of medicine at the patient level. Their perception is thus shaped by the direct impact on the patient. If medicines are not available, they sometimes address patients’ frustration without the authority to change prescriptions or solve supply problems. These differences do not reflect disagreements about the existence of a shortage, but rather differing experiences of failures in governance in supply, clinical practice and patient care. Understanding these interconnected levels is essential to develop effective solutions.

Medicine shortage has various effects on different stakeholders, especially patients. It results in inadequate treatment, the use of substitutions that might delay patient care, prolonged hospitalisation, and increased re-admission rates [45,46]. Moreover, due to medicine shortages, patients’ out-of-pocket costs are increased because patients have to purchase costly medicines that are not provided by the public health system [47]. Atif et al. [6] highlighted that up to 25% of Pakistani people live below the poverty line and cannot afford out-of-pocket expenses. Out-of-pocket spending is an important indication of financial security and specifies the private involvement stance required for health funding [48].

Evidence in South Africa has confirmed a similar situation to the results reported by low-income Pakistanis, that access to medicines remains an important obstacle for people living below or near the poverty line, particularly when public sector availability is lacking. Data from the general household survey and the national income dynamics study show that a significant proportion of South Africans rely exclusively on the public health sector and do not have the financial capacity to buy medicines when stock shortages occur [30,48].

This study supplements this literature by foregrounding the perspectives of health professionals, who often witness patients not being able to afford drugs when the public sector’s supply fails, particularly in resource-limited provinces such as Eastern Cape. The Pakistani and global studies have affirmed that medical shortages increase drug prices, requiring patients to buy expensive alternatives from private pharmacies, and aggravate financial difficulties [5,6,46]. Mostly, hospital medical professionals showed a greater sensitivity to increased financial burdens for patients resulting from a shortage of medicines. In the study, pharmacists were the most likely group to report medicine shortages, reflecting their direct experience with medicines that were unavailable and forced to be replaced.

The common perception of treatment delays in all populations reveals a widespread recognition of this systemic problem in clinical and other health systems. The lack of affordable medicines is the main cause of the widespread cost of patient care and is a major obstacle to universal coverage of healthcare. This problem is widespread and affects both low-income and high-income countries and continues to pose a complex and costly challenge to the health system [49].

The groundbreaking data to which this study contributes include the perception of various medical professionals regarding the shortage of medicine. In the same public health system in South Africa, different healthcare professionals are accessible at various levels of care across one province. These facilities serve the majority of the population and cannot absorb costs from the outside in private. In this context, it has been demonstrated that health professionals (pharmacologists, nurses and medical officials) have different experiences. Pharmacists reported greater exposure to the clinical, financial and emotional consequences of medicine shortages. This may indicate their central role in drug management and procurement coordination. They are also the last contact point with health facilities’ patients.

By empirically linking medicine shortages and interprofessional relationships, the study extends the current evidence beyond patients and the health system. This includes the overall organisational governance and other workforces with more procurement and financial authority than pharmacists. This misalignment between authority and control reframes medicine shortage as a systemic governance challenge, rather than mere procurement failure.

Based on the healthcare professionals’ perceptions of patient financial distress during medicine shortages, the study adds to the grounded literature on medicine affordability and health system equity. It highlights the policy gap that, in a setting where public health sector medicine availability is the only primary access to healthcare, patients lack financial protection. This indicates that the pre-defined public–private continuity arrangements are a policy imperative in a health system that recognises access to essential medicines as a fundamental human right. This responsibility cannot be shifted to patients through out-of-pocket payments.

The findings of the study highlight that the experiences of medicine shortages are different among the health professionals’ categories. This implies that the interventions cannot be focused on one category or at the facility level. The policy response must focus on all governance levels of healthcare from the clinic to the highest level of decision making. The healthcare professionals’ mandate involves developing the standard treatments guidelines and essential medicines lists and selection, procurement and distribution of medicines. These mandates are implemented through the established Pharmacy and Therapeutics Committees. Policy decisions entail the inclusion of certain financial authorities that will make healthcare professionals accountable for procurement of medicines and timely payment of suppliers. This will ensure that medicines are available. This will also be critical in their responsiveness to shortages that are related to manufacturing and the communication to all levels will be channeled through the PTC, including the available substitutions.

## 5. Limitation

Despite its strong findings, the research has some limitations. Firstly, a cross-sectional design provides a snapshot of perceptions at a single point in time and cannot establish causality. Secondly, the study was carried out in a South African province (Eastern Cape), limiting its scope to other states and countries; however, the identified problems could represent wider resource-limited public health challenges. Thirdly, the use of self-administered questionnaires is practical, but there are social needs biases, and respondents may provide expected answers. Finally, these studies are limited by their exclusive focus on health professionals. A more comprehensive understanding of the impact of medicine shortages requires the inclusion of patient and hospital administrator perspectives.

## 6. Recommendations

Strengthening the management of the pharmaceutical supply chain is essential and requires strict adherence to the WHO framework for good medicine management. These include improving the transparency of procurement and procurement processes, improving inventory management systems and real-time tracking, and establishing a national self-sufficiency warning system for future shortages. Furthermore, establishing and disseminating clear standard operating procedures (SOPs) will strengthen transparency. Standard operating procedures must clearly outline protocols and procedures for the substitution of treatments, the communication channels between pharmacists, medical officers, and patients, procedures for assessing patient referrals to mitigate clinical risks, and unified guidelines for managing drug stocks. It is important to improve interprofessional cooperation to promote a culture of teamwork and common responsibility. Regular, structured interprofessional meetings should be held at the facility level to jointly plan and manage shortages and reduce the burdens and blame that are often mainly transferred to pharmacists. The review of current health funding policies to protect patients from unexpected expenses during a shortage should be undertaken regularly. This includes expanding the coverage of the essential medicine list or creating a provisional fund to cover the costs of alternative medicines in the public system.

## 7. Future Research

Further qualitative research is needed to investigate patients’ personal experiences and the root causes of supply chain failures from the perspectives of policymakers and suppliers. Long-term studies to track the impact of specific interventions over time are needed.

## 8. Conclusions

In conclusion, the negative impact of the shortage of medicines is felt worldwide, but this study provides specific, quantitative evidence that these effects are differently perceived in South African healthcare teams. Increased concern among pharmacists reflects their crucial and strained role in managing this crisis. The study clearly shows that South African public healthcare professionals consider the shortage of medicines to be a major problem that has a significant impact on patient safety, clinical outcomes, and health system efficiency. The differences in perception across professional categories, particularly the growing concerns among pharmacists, highlight the need for specific and joint support in crisis management. To solve this multifaceted problem, a coordinated effort is needed that combines strong supply chain management, strong leadership, clear policies, and commitment to interprofessional teamwork. Reduced medicine shortages are not only a logistical challenge but also a basic prerequisite for achieving equitable access to healthcare and the goal of universal health coverage in South Africa.

## Figures and Tables

**Table 1:** Distribution of respondents by gender, profession, level of care, age, and years of service (N = 394).

Variable			Category			n	%
Gender			Female			281	71.3
			Male			113	28.7
Profession			Medical Officers			77	19.5
			Professional Nurses			217	55.1
			Pharmacists			100	25.4
Level of Care			CHC			67	17.0
			Clinic			128	32.5
			Hospital			199	50.5
Variable	Minimum	LQ	Median	Mean	Mode	UQ	Maximum
Age (years)	28.0	41.0	47.0	46.9	43.0	53.0	65.0
Years of Service	2.0	13.0	19.0	19.1	21.0	23.0	40.0

Abbreviations: CHC, community health centre; LQ, lower quartile; UQ, upper quartile.

**Table 2:** Distribution of healthcare professionals’ opinions on the impact of medicine shortages (N = 394).

Variable	Disagree n (%)	Neutral n (%)	Agree n (%)
Stakeholder impact	8 (2.03)	18 (4.57)	368 (93.41)
Extra costs for patients	14 (3.55)	64 (16.24)	316 (80.20)
Out-of-pocket costs	59 (14.97)	143 (36.29)	192 (48.73)
Errors in treatment and dispensing	106 (26.90)	136 (34.52)	152 (38.57)
Denied or delayed treatment	23 (5.84)	93 (23.60)	278 (70.56)
Provider stress	1 (0.25)	12 (3.05)	381 (96.70)
Physicians forced to ration or use alternatives	12 (3.05)	88 (22.34)	294 (74.62)
Compromised health outcomes	29 (7.36)	101 (25.63)	264 (67.00)

Source: Self-designed.

**Table 3:** Bivariate analysis of associations between respondents’ characteristics and perceived impacts of medicine shortages.

Variable	Age		Years of Service		Gender		Profession		Level	
	KW	*p*-Value	KW	*p*-Value	χ^2^	*p*-Value	χ^2^	*p*-Value	χ^2^	*p*-Value
Stakeholder impact	1.6	0.446	0.7	0.723	1.4	0.496	12.4	0.015	3.8	0.432
Extra costs for patients	2.8	0.246	2.5	0.290	4.3	0.117	33.8	0.001	46.5	0.001
Out-of-pocket costs	1.6	0.453	0.7	0.702	2.8	0.246	56.3	0.001	41.4	0.001
Errors in treatment/dispensing	15.2	0.001	13.9	0.001	1.8	0.406	29.6	0.001	10.2	0.037
Denied/delayed treatment	8.2	0.017	7.1	0.029	1.5	0.466	18.3	0.001	5.8	0.216
Provider stress	4.1	0.131	5.0	0.083	0.5	0.783	4.9	0.302	2.6	0.627
Medical officers forced to ration/use alternative drugs	3.2	0.200	3.7	0.161	3.1	0.211	6.8	0.145	3.2	0.527
Compromised health outcomes	2.7	0.257	4.5	0.104	0.6	0.725	24.9	0.001	8.0	0.090

Abbreviations: KW, Kruskal–Wallis test; χ^2^, Chi-square test.

**Table 4:** Out-of-pocket costs to patients.

Odds Ratio Estimates		Crude Odds Ratios			Adjusted Odds Ratios		
Effect	Predictor	Point Estimate	95% Wald		Point Estimate	95% Wald	
			Confidence Limits			Confidence Limits	
Gender Female vs. Male	Neutral	0.781	0.381	1.603	1.118	0.505	2.477
Gender Female vs. Male	Agree	0.593	0.299	1.177	1.231	0.555	2.729
Profession Medical Officer vs. Pharmacist	Neutral	0.667	0.168	2.638	0.68	0.17	2.717
Profession Medical Officer vs. Pharmacist	Agree	0.794	0.219	2.885	0.843	0.23	3.095
Profession Nurse vs. Pharmacist	Neutral	0.37	0.134	1.021	0.612	0.192	1.949
Profession Nurse vs. Pharmacist	Agree	0.105 *	0.039	0.28	0.187 *	0.061	0.578
Level CHC vs. Hospital	Neutral	0.533	0.209	1.363	0.604	0.227	1.61
Level CHC vs. Hospital	Agree	0.379 *	0.154	0.93	0.57	0.218	1.488
Level Clinic vs. Hospital	Neutral	0.356 *	0.174	0.728	0.446	0.183	1.083
Level Clinic vs. Hospital	Agree	0.121 *	0.059	0.249	0.349 *	0.141	0.867

* Statistically significant at *p* < 0.05 (95% CI does not include 1.00).

**Table 5:** Errors in treatment, prescription, dispensing and administration of medicines.

Odds Ratio Estimates		Crude Odds Ratios			Adjusted Odds Ratios		
Effect	Predictor	Point Estimate	95% Wald		Point Estimate	95% Wald	
			Confidence Limits			Confidence Limits	
Gender Female vs. Male	Neutral	0.680	0.384	1.202	0.869	0.46	1.641
Gender Female vs. Male	Agree	0.824	0.468	1.451	0.964	0.508	1.828
Profession Medical Officer vs. Pharmacist	Neutral	0.504	0.201	1.267	0.484	0.191	1.224
Profession Medical Officer vs. Pharmacist	Agree	0.321 *	0.131	0.789	0.335 *	0.135	0.832
Profession Nurse vs. Pharmacist	Neutral	0.257 *	0.119	0.556	0.165 *	0.067	0.404
Profession Nurse vs. Pharmacist	Agree	0.154 *	0.073	0.326	0.122 *	0.052	0.286
Level CHC vs. Hospital	Neutral	0.528	0.259	1.076	0.772	0.358	1.662
Level CHC vs. Hospital	Agree	0.494 *	0.253	0.967	0.691	0.332	1.439
Level Clinic vs. Hospital	Neutral	0.895	0.507	1.578	2.442 *	1.157	5.155
Level Clinic vs. Hospital	Agree	0.507 *	0.286	0.901	1.352	0.654	2.793

* Statistically significant at *p* < 0.05 (95% CI does not include 1.00).

**Table 6:** Delayed or denied treatment, prolonged hospitalisation and adverse medicine reactions.

Odds Ratio Estimates		Crude Odds Ratios			Adjusted Odds Ratios		
Effect	Predictor	Point Estimate	95% Wald		Point Estimate	95% Wald	
			Confidence Limits			Confidence Limits	
Gender Female vs. Male	Neutral	0.515	0.16	1.654	0.54	0.157	1.86
Gender Female vs. Male	Agree	0.503	0.166	1.525	0.619	0.191	2.01
Profession Medical Officer vs. Pharmacist	Neutral	1.6	0.249	10.268	1.383	0.212	9.021
Profession Medical Officer vs. Pharmacist	Agree	1.555	0.276	8.762	1.471	0.258	8.387
Profession Nurse vs. Pharmacist	Neutral	1.035	0.304	3.524	1.395	0.345	5.644
Profession Nurse vs. Pharmacist	Agree	0.389	0.127	1.197	0.385	0.107	1.386
Level CHC vs. Hospital	Neutral	0.52	0.156	1.738	0.489	0.139	1.718
Level CHC vs. Hospital	Agree	0.4	0.132	1.213	0.528	0.166	1.678
Level Clinic vs. Hospital	Neutral	0.78	0.272	2.236	0.717	0.205	2.512
Level Clinic vs. Hospital	Agree	0.492	0.183	1.323	1.046	0.321	3.404

**Table 7:** Medicine shortages lead to compromised health outcomes.

Odds Ratio Estimates		Crude Odds Ratios			Adjusted Odds Ratios		
Effect	Predictor	Point Estimate	95% Wald		Point Estimate	95% Wald	
			Confidence Limits			Confidence Limits	
Gender Female vs. Male	Neutral	1.099	0.434	2.781	0.888	0.318	2.481
Gender Female vs. Male	Agree	0.892	0.379	2.099	0.912	0.353	2.354
Profession Medical Officer vs. Pharmacist	Neutral	0.105 *	0.019	0.591	0.109 *	0.019	0.622
Profession Medical Officer vs. Pharmacist	Agree	0.166 *	0.035	0.797	0.177 *	0.036	0.861
Profession Nurse vs. Pharmacist	Neutral	0.427	0.091	2.003	0.342	0.062	1.891
Profession Nurse vs. Pharmacist	Agree	0.177 *	0.04	0.785	0.128 *	0.025	0.665
Level CHC vs. Hospital	Neutral	3.705	0.773	1.753	3.757	0.736	19.174
Level CHC vs. Hospital	Agree	2.59	0.574	11.684	3.461	0.719	16.664
Level Clinic vs. Hospital	Neutral	1.455	0.603	3.511	1.512	0.483	4.73
Level Clinic vs. Hospital	Agree	0.778	0.344	1.762	1.624	0.55	4.94

* Statistically significant at *p* < 0.05 (95% CI does not include 1.00).

## Data Availability

The data presented in this study are available on request from the corresponding author due to privacy and legal implications.

## Abbreviations

ANOVA	Analysis of Variance
CHC	Community Health Centre
EML	Essential Medicines List
NMRA	National Medicine Regulatory Authority
PHC	Primary Healthcare
POPIA	Protection of Personal Information Act
SD	Standard Deviation
SDG	Sustainable Development Goal
SE	Standard Error
SPSS	Statistical Package for the Social Sciences
STG	Standard Treatment Guidelines
WHO	World Health Organization
